# Improved clinical outcomes when maintaining a neutral joint line obliquity at high tibial osteotomy and subsequent total knee arthroplasty

**DOI:** 10.1002/jeo2.70612

**Published:** 2026-01-13

**Authors:** Adit R. Maniar, Nicola D. Mackay, Lyndsay Somerville, Robert Litchfield, Brent A. Lanting, Alan M. J. Getgood

**Affiliations:** ^1^ London Health Sciences Center London Ontario Canada

**Keywords:** high tibial, joint line obliquity, osteotomy, outcomes, total knee arthroplasty

## Abstract

**Purpose:**

The primary aim was to study the impact of joint line obliquity (JLO) on clinical outcomes and survivorship in patients undergoing total knee arthroplasty (TKA) after a previous high tibial osteotomy (HTO). The secondary aim was to study how maintaining neutral JLO at both HTO and TKA affected clinical outcomes of TKA.

**Methods:**

A retrospective review of patients undergoing TKA following valgus‐producing HTO, having a minimum 1‐year follow‐up, was performed. Using the coronal plane alignment of the knee (CPAK) classification, three groups of JLO were formed: distal JLO (<177°), neutral JLO (177–183°) and proximal JLO (>183°). Clinical outcomes were assessed using the Western Ontario and McMaster University Osteoarthritis Index (WOMAC). The level of significance was 0.05.

**Results:**

The study included 110 TKA (mean follow‐up: 5.8 years). Prevalence of a proximal JLO post‐TKA was higher (*p* < 0.05) in those with a proximal JLO pre‐TKA (40%) as compared to those with a neutral JLO pre‐TKA (13%). The odds ratio of having a proximal JLO post‐TKA was 4 (95% confidence interval: 1.4–11.1, *p* < 0.05) in those having a proximal JLO pre‐TKA. Revision rate in the proximal JLO post‐TKA, neutral JLO post‐TKA and distal JLO post‐TKA groups was 20%, 8% and 4% respectively, with no statistical difference (*p* > 0.05). Post‐TKA, the stiffness, function and total WOMAC were significantly better (*p* < 0.05) in patients with a neutral JLO pre‐TKA and neutral JLO post‐TKA as compared to those with a proximal JLO pre‐TKA and proximal JLO post‐TKA or a proximal JLO pre‐TKA and neutral JLO post‐TKA.

**Conclusion:**

While a proximal JLO after conversion TKA did not show a statistically higher revision rate, maintaining a neutral JLO during HTO and at the time of subsequent TKA was associated with higher post‐operative clinical scores. However, further research in different and larger populations is needed to confirm these findings.

**Level of Evidence:**

Level IV.

**Abbreviations**
DLO: double level osteotomy
FJS: Forgotten Joint Score
HKA: hip‐knee‐ankle
HTO: high tibial osteotomy
JLO: joint line obliquity
LCW: lateral closing wedge
LDFA: lateral distal femoral angle
MOW: medial opening wedge
MPTA: medial proximal tibial angle
OA: osteoarthritis
OR: odds ratio
ROM: range of motion
TKA: total knee arthroplasty
WOMAC: Western Ontario and McMaster University Osteoarthritis Index

## INTRODUCTION

There has been a steady growth in osteoarthritis (OA) ARM burden among those 15–49 years of age [1], with the medial compartment being the most commonly involved [21]. A high tibial osteotomy (HTO) is an effective surgical option to preserve the joint, relieve symptoms, improve function and delay the need for a total knee arthroplasty (TKA) [5, 23]. A valgus‐producing HTO has shown excellent results and survivorship in managing early isolated medial compartment OA in the young patient [17]. In the event of progressive, symptomatic OA, a subsequent conversion TKA can be considered as the definitive surgical option. The outcomes and survivorship of conversion TKA following a HTO have been shown to be equivalent to a primary TKA [22].

Overall alignment following HTO does not seem to affect outcomes or survivorship of conversion TKA [11, 19]. However, these studies have not taken the joint line obliquity (JLO) into consideration. JLO is important, as by increasing the JLO, the shear forces on the tibial cartilage increase [16], predisposing it to faster wear. Song et al. [20] found poorer clinical outcomes when the JLO was more than 4° and poorer radiological outcomes when the JLO was more than 6° after a medial opening wedge (MOW) HTO. Similarly, by maintaining JLO during a TKA, patients had a better Forgotten Joint Score (FJS) and range of motion (ROM) [6]. Thus, JLO is an important factor that can affect both HTO and TKA outcomes.

The primary aim was to study the impact of abnormal JLO after conversion TKA on clinical outcomes and survivorship. The secondary aim was to study the effect of maintaining a neutral JLO both at HTO and TKA on clinical outcomes of conversion TKA. We hypothesized that JLO post‐TKA and maintaining a neutral JLO both during HTO and TKA would not affect outcomes post conversion TKA.

## METHODS

After research ethics board approval (REB 124314), a retrospective review of a prospectively collected institutional database at a single tertiary care centre was performed. The institutional database collects data on all arthroplasty cases (both hip and knee) performed locally by all arthroplasty surgeons at our institute. These data are collected prospectively and entered into the database on an ongoing basis. Data is collected preoperatively, intraoperatively and post‐operatively. This database has existed since the early 2000s and collects post‐operative outcomes annually for those returning for routine visits. The inclusion criteria for this study were all patients undergoing conversion TKA following an osteotomy between 1 January 2007 and 31 December 2022, with a minimum of 1 year follow‐up and having pre‐TKA and post‐TKA long leg alignment radiographs available. We excluded all patients who underwent conversion TKA following an osteotomy other than a valgus‐producing HTO. We identified 13,671 TKAs in the database during this time period, of which 195 TKAs met the inclusion criteria. Of these, 85 TKAs were excluded based on exclusion criteria. Thus, we were left with 110 TKAs for final analysis.

All patients underwent TKA using a mechanical alignment philosophy. Preoperative demographics, implant type, method of fixation and presence of patella resurfacing were recorded. Clinical outcomes were assessed using the Western Ontario and McMaster University Osteoarthritis Index (WOMAC), a validated outcome tool [13], which was collected annually as per institutional protocol. The score was inverted on a scale of 100 such that higher score represents a better score. Survivorship was assessed using revision surgery for any reason as an endpoint. All patients had pre‐TKA and post‐TKA long leg alignment radiographs performed at a single center utilizing the same protocol.

**Figure 1:**
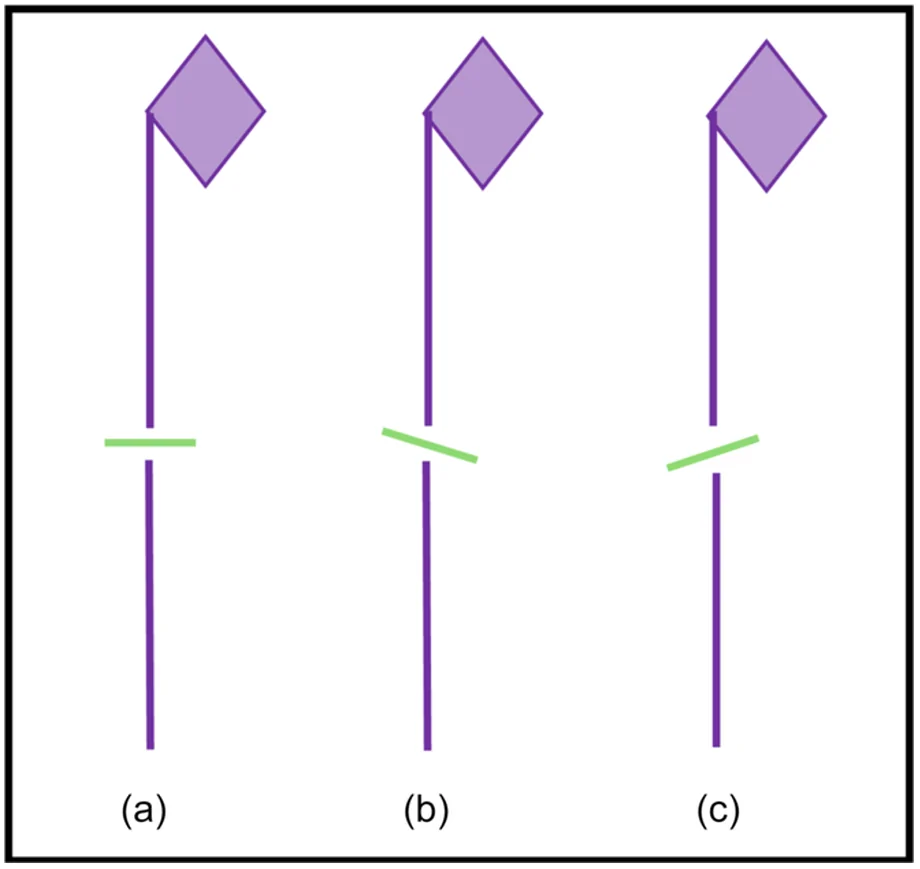
Representative line figure showing three types of joint line obliquity (JLO) based on coronal plane assessment of the knee (CPAK) classification. The purple line represents the limb alignment. The green line represents the joint line. (a) Neutral JLO—joint line parallel to the ground. (b) Distal JLO—joint line pointing downwards towards the midline. (c) Proximal JLO—joint line pointing upwards towards the midline.

JLO was defined using the coronal plane alignment of the knee (CPAK) classification [15]. As per this classification, JLO is defined as the sum of the mechanical medial proximal tibial angle (MPTA) and the mechanical lateral distal femoral angle (LDFA). Patients are then categorized into three categories: neutral (JLO = 177–183°), proximal (JLO > 183°) and distal (JLO < 177°) (Figure 1). A valgus‐producing HTO (MOW or lateral closing wedge [LCW]) would predispose a patient to having a proximal JLO by increasing the MPTA. Pre‐TKA and post‐TKA hip‐knee‐ankle (HKA) angles, MPTA and LDFA were measured using mediCAD (Version 7.0) software. A positive HKA angle was used for a varus deformity and a negative HKA angle for a valgus deformity. Two independent observers (AM*, NM*) made 50 measurements to measure inter‐observer and intra‐observer reliability. For intra‐observer reliability, repeat measurements were made after a period of 6 weeks. The intraclass coefficients were excellent (>0.9) for all measurements (Table 1). Therefore, final results were analyzed using measurements from a single observer (*AM*).

**Table 1:** Inter‐observer and intra‐observer reliability.

	Inter‐observer reliability	Intra‐observer reliability
	ICC (95% CI)	ICC (95% CI)
HKA	0.997 (0.995–0.998)	0.992 (0.986–0.995)
MPTA	0.924 (0.870–0.956)	0.923 (0.868–0.955)
LDFA	0.916 (0.849–0.953)	0.922 (0.866–0.955)

Abbreviations: CI, confidence interval; HKA, hip‐knee‐ankle angle; ICC, intraclass correlation; LDFA, lateral distal femoral angle; MPTA, medial proximal tibial angle.

### Statistical analysis

Statistical analysis was performed using IBM SPSS Statistics (V30). Descriptive statistics were used to describe the demographics of the sample, including means, standard deviations and ranges as well as frequencies and proportions where applicable. The proportion of those falling into proximal, neutral and distal JLO post‐TKA was calculated in relation to their pre‐TKA JLO category. The odds ratio (OR) and corresponding 95% confidence intervals (CIs) were then calculated based on these categorizations. Analysis of variance was performed to determine demographic and clinical differences in the patients with proximal, neutral or distal JLO based on their post‐TKA radiographs. Revision rates were calculated, and chi‐squared analysis was performed. To study the effect of maintaining a neutral JLO at both HTO and TKA, the patients were divided into three subgroups: (a) neutral JLO pre‐TKA and neutral JLO post‐TKA, (b) proximal JLO pre‐TKA and neutral JLO post‐TKA, and (c) proximal JLO pre‐TKA and proximal JLO post‐TKA. Analysis of covariance was performed to determine demographic and clinical differences between these groups. The level of significance was set at 0.05. To confirm the clinical relevance of any statistical differences in the WOMAC scores, the minimum clinically important difference (MCID) established by Clement et al. [7], which is 10 points for the total WOMAC and 8 and 9 points for the stiffness and function sub‐scores, respectively, was used.

## RESULTS

**Figure 2:**
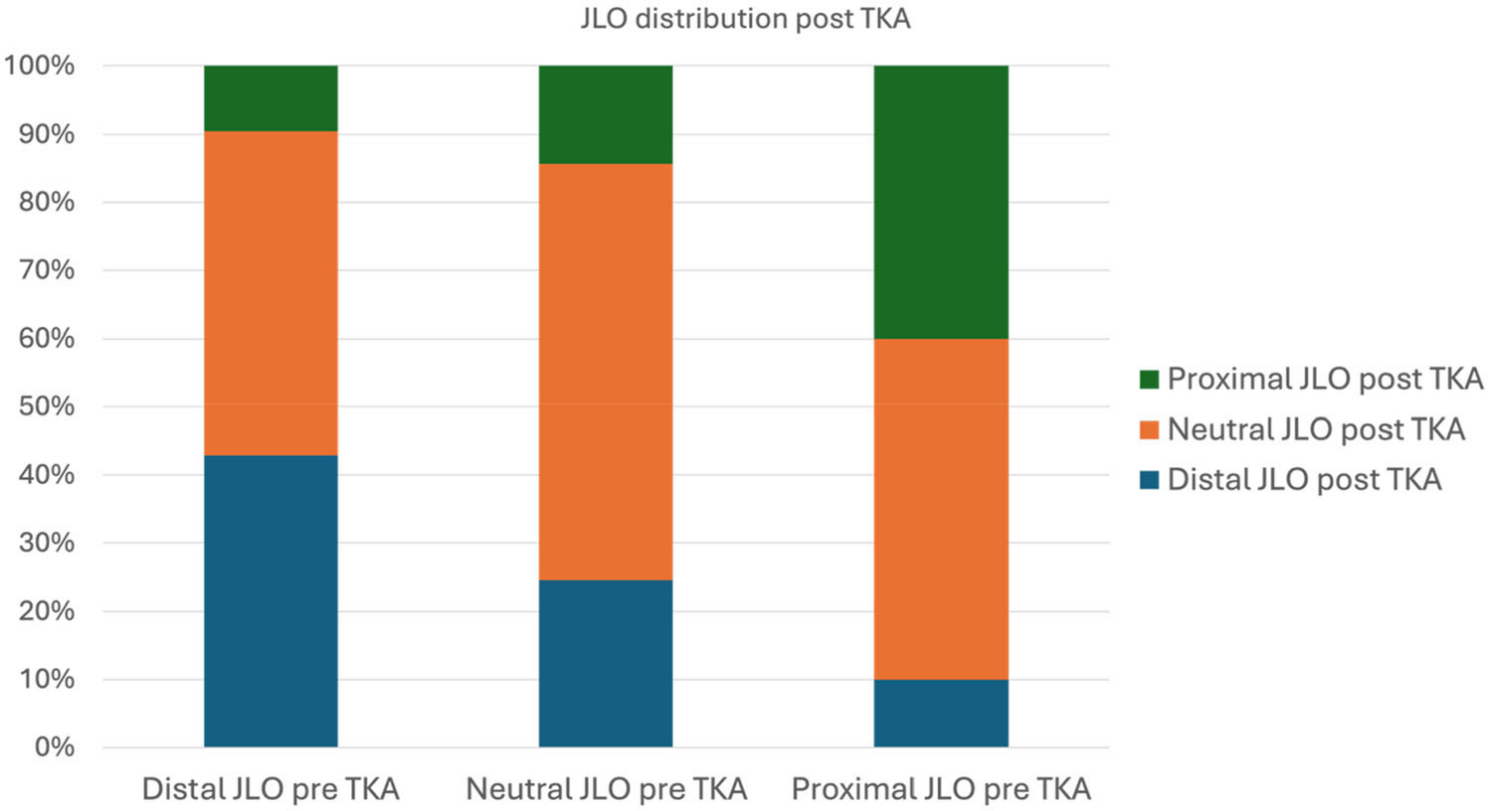
Joint line obliquity (JLO) achievement rates post conversion total knee arthroplasty (TKA).

There were 110 eligible TKA patients for this study. Of these, 98 underwent an MOW HTO while the remaining patients underwent an LCW HTO. The mean age was 59 years (range: 44–79 years), and the mean follow‐up post‐TKA was 5.8 years (range: 1–16 years). Preoperative demographics, implant type, method of fixation and presence of patella resurfacing are summarized in Table 2. The prevalence of a proximal JLO post‐TKA was higher in those with a proximal JLO pre‐TKA (40%) as compared to those with a neutral JLO pre‐TKA (13%) (*p* = 0.004) (Figure 2). The OR of having a proximal JLO post‐TKA was 4 (95% CI: 1.4–11.1; *p* = 0.008) in those with a proximal JLO pre‐TKA as compared to those with a neutral JLO pre‐TKA.

**Table 2:** Preoperative demographics of the entire cohort.

Age in years (mean ± SD, range)	59 ± 8, 44–79
BMI in kg/m^2^ (mean ± SD)	33 ± 7
Follow‐up in years (mean, range)	5.8, 1–16
Female gender, *N* (%)	34 (31)
Constraint, *N* (%)	
PS	93 (84)
CS	14 (13)
VVC	3 (3)
Patella resurfaced, *N* (%)	49 (45)
Cemented fixation, *N* (%)	105 (96)

Abbreviations: BMI, body mass index; CS, cruciate substituting; N, number; PS, posterior stabilized; SD, standard deviation; VVC, varus–valgus constraint.

### Post‐TKA JLO and clinical outcomes

**Figure 3:**
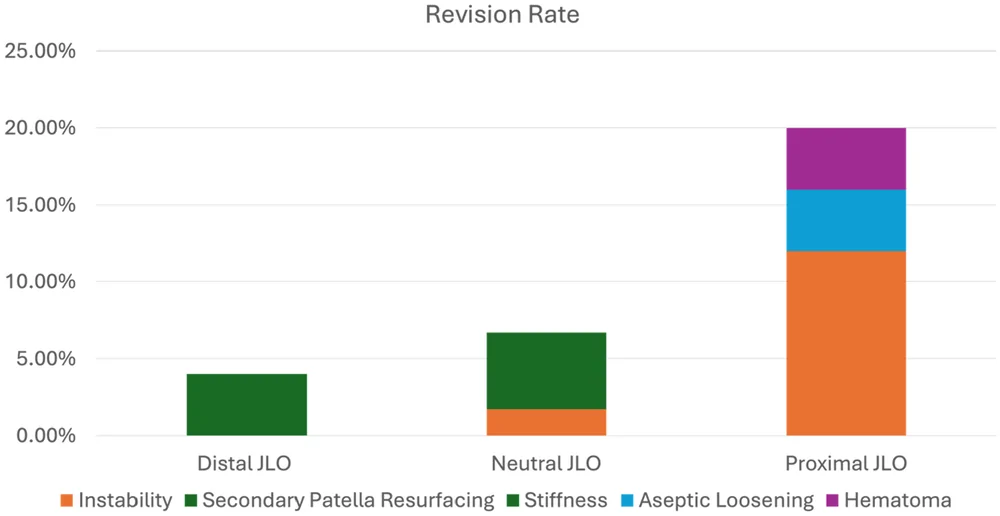
Comparison of survivorship between groups based on joint line obliquity (JLO) post conversion total knee arthroplasty.

There were no differences (*p* > 0.05) in preoperative age, sex, type of HTO, pre‐TKA HKA and WOMAC score between the three groups (Table 3). The preoperative BMI was higher (*p* = 0.03) in the distal JLO group (Table 3). The level of constraint used, the method of fixation and the incidence of patella resurfacing were comparable (*p* > 0.05) between the three groups (Table 3). The pre‐TKA LDFA was higher (*p* < 0.01) in the proximal JLO group. The post‐TKA MPTA and LDFA were higher (*p* < 0.01) in the proximal JLO group (Table 3). The mean follow‐up was 5.6, 6.2 and 5.2 years in the distal, neutral and proximal JLO groups, respectively, with no statistical difference (*p* = 0.57) between the three groups (Table 3). The post‐TKA HKA and WOMAC were comparable between the three groups (Table 3). Interestingly, despite no statistical difference (*p* > 0.05), the revision rate was 20% in the proximal JLO group as compared to 8.3% and 4% in the neutral and distal JLO groups, respectively (Figure 3).

**Table 3:** Comparison between groups based on post‐TKA JLO.

	JLO distal	JLO neutral	JLO proximal	*p*
*N*	25	60	25	
Age in years (range)	58 (44–74)	57 (44–75)	62 (5179)	0.05
Sex (% women)	32.0%	33.3%	24.0%	0.69
BMI (kg/m^2^)	36.3 ± 7.5	32.4 ± 6.7	31.9 ± 5.9	0.03
Constraint used				
CR/CS	16.0%	10.0%	16.0%	0.16
PS	80.0%	90.0%	76.0%	
VVC	4.0%	0.0%	8.0%	
Patella resurfacing (% yes)	40.0%	43.3%	52.0%	0.67
Fixation (% cemented)	92.0%	95.0%	100.0%	0.41
MOW/LCW (% MOW)	92.0%	81.7%	72.0%	0.19
Pre‐TKA HKA (°)	1.0 ± 6.3	0.8 ± 5.1	1.8 ± 4.9	0.75
Pre‐TKA LDFA (°)	87.9 ± 1.8	89.1 ± 2.0	90.8 ± 2.4	<0.01
Pre‐TKA MPTA (°)	91.1 ± 3.7	92.0 ± 3.7	93.5 ± 3.6	0.08
Post‐TKA HKA (°)	1.3 ± 3.7	‐0.4 ± 3.2	0.6 ± 3.1	0.06
Post‐TKA LDFA (°)	88.4 ± 1.9	89.8 ± 1.4	92.7 ± 2.1	<0.01
Post‐TKA MPTA (°)	87.2 ± 1.8	90.3 ± 1.9	92.2 ± 1.4	<0.01
Mean follow‐up in years (range)	5.6 (1–16)	6.2 (1–16)	5.2 (1–12)	0.57
Pre‐TKA WOMAC (mean ± SD)				
Pain	44.6 ± 20.6	39.6 ± 17.2	47.1 ± 15.1	0.23
Stiffness	36.3 ± 25.0	40.9 ± 21.6	37.5 ± 12.8	0.66
Function	46.3 ± 19.5	43.1 ± 14.9	53.5 ± 14.2	0.05
Total	42.8 ± 19.3	41.2 ± 15.3	47.4 ± 12.3	0.31
Post‐TKA WOMAC (mean ± SD)				
Pain	72.6 ± 27.3	73.6 ± 22.0	77.6 ± 19.5	0.70
Stiffness	68.0 ± 20.8	61.5 ± 24.8	65.0 ± 25.8	0.51
Function	73.3 ± 21.6	72.4 ± 21.8	73.5 ± 20.9	0.97
Total	71.9 ± 21.9	70.6 ± 21.2	73.5 ± 19.6	0.85

Abbreviations: BMI, body mass index; CR, cruciate retaining; CS, cruciate substituting; HKA, hip‐knee‐ankle angle; JLO, joint line obliquity; LCW, lateral closing wedge; LDFA, lateral distal femoral angle; MOW, medial open wedge; MPTA, medial proximal tibial angle; N, number; PS, posterior stabilized; SD, standard deviation; TKA, total knee arthroplasty; VVC, varus–valgus constraint; WOMAC, Western Ontario and McMaster University Osteoarthritis Index.

### Maintaining JLO and clinical outcomes

To study the effect of maintaining a neutral JLO at both HTO and TKA, the patients were divided into three subgroups as described earlier. Pre‐TKA, there were no differences (p > 0.05) in sex, type of HTO, HKA and WOMAC score between the three groups (Table 4). There was a statistical difference in age (*p* = 0.03), with age being highest in the group with proximal JLO pre‐ and post‐TKA. The level of constraint used, method of fixation, incidence of patella resurfacing and follow‐up were comparable (*p* > 0.05) between the three groups (Table 4). There was a statistical difference in post‐TKA HKA alignment (*p* = 0.04), but this was not clinically relevant (−1.1°, 0.1° and 1.3°, respectively) (Table 4). Post‐operatively, the mean total WOMAC and its stiffness and function subcomponents were significantly better (*p* < 0.05) in the group which had a neutral JLO both pre‐ and post‐TKA (Table 4).

**Table 4:** Subgroup analysis: comparison based on maintaining JLO pre‐ and post‐TKA.

	Neutral JLO Pre‐TKA + Post‐TKA	Proximal JLO Pre‐TKA + Neutral JLO Post‐TKA	Proximal JLO Pre‐TKA + Post‐TKA	*p*
*N*	30	20	16	
Age in years (range)	57 (46–68)	57 (44–67)	62 (52–79)	0.03
Sex (% women)	33.3%	30.0%	25.0%	0.94
BMI (kg/m^2^)	32.4 ± 7.2	32.8 ± 5.9	32.9 ± 6.5	0.97
Constraint used				
CR/CS	10.0%	15.0%	18.8%	0.44
PS	90.0%	85.0%	75.0%	
VVC	0.0%	0.0%	6.3%	
Patella resurfacing (% yes)	43.3%	40.0%	43.8%	0.97
Fixation (% cemented)	96.7%	90.0%	100.0%	0.45
MOW/LCW (% MOW)	80.0%	90.0%	75.0%	0.46
Mean follow‐up in years (range)	6.2 (1–14)	6.5 (1–16)	5.0 (1–10)	0.55
Pre‐TKA HKA (°)	−0.2 ± 4.1	−0.4 ± 3.6	0.1 ± 4.5	0.94
Post‐TKA HKA (°)	−1.1 ± 3.1	0.1 ± 2.6	1.3 ± 3.1	0.04
Pre‐TKA WOMAC (mean ± SD)				
Pain	36.1 ± 18.0	37.9 ± 10.5	43.9 ± 14.3	0.36
Stiffness	40.3 ± 21.5	40.6 ± 17.8	34.6 ± 14.6	0.64
Function	42.6 ± 15.8	37.8 ± 11.5	50.2 ± 13.4	0.09
Total	39.4 ± 16.6	38.4 ± 9.7	44.3 ± 11.9	0.51
Post‐TKA WOMAC (mean ± SD)				
Pain	78.8 ± 19.6	66.0 ± 20.5	70.3 ± 19.9	0.08
Stiffness	67.9 ± 22.2	51.3 ± 24.3	51.6 ± 20.9	0.02
Function	80.4 ± 17.8	59.6 ± 20.1	66.0 ± 20.7	<0.01
Total	77.1 ± 18.5	60.5 ± 18.4	64.8 ± 18.4	<0.01

Abbreviations: BMI, body mass index; CR, cruciate retaining; CS, cruciate substituting; HKA, hip‐knee‐ankle angle; JLO, joint line obliquity; LCW, lateral closing wedge; LDFA, lateral distal femoral angle; MOW, medial open wedge; MPTA, medial proximal tibial angle; N, number; PS, posterior stabilized; SD, standard deviation; VVC, varus–valgus constrained; WOMAC, Western Ontario and McMaster University Osteoarthritis Index.

## DISCUSSION

This study found that patients with a proximal JLO pre‐TKA had a significantly higher chance (OR = 4) of having a proximal JLO post‐TKA. JLO grouping post‐TKA did not seem to affect clinical outcomes. Despite no statistical difference, patients with a proximal JLO had a higher incidence of revision surgery (12%–16%), with instability being the most common indication for revision in this group. Interestingly, patients having a neutral JLO pre‐TKA (i.e., after the HTO) and neutral JLO post‐TKA had significantly higher WOMAC scores post‐TKA.

Patients who had a proximal JLO post‐TKA had a significantly higher LDFA pre‐TKA. By ignoring the femoral deformity and performing only an HTO in patients with a femoral varus deformity, the surgeon needs to increase the MPTA (valgus deformity of the tibia) to achieve an appropriate correction. At the time of subsequent TKA, to achieve neutral mechanical alignment in such patients, the resultant bony cut on the lateral side may be inadequate, forcing surgeons to make a slight valgus tibial cut or to increase the depth of the bony cut. On the femoral side, the uncorrected femoral varus may predispose to a varus distal femoral cut with standard intramedullary techniques. These alterations in bony cuts can lead to an increased JLO post‐TKA. However, at the time of index osteotomy, a double‐level osteotomy (DLO) [8] can address both the femoral and tibial deformities, not only maintaining a neutral JLO, but also allowing for more appropriate bony cuts at the time of TKA.

The revision rate was noted to be higher (20% vs. 4.0% and 8.3%) in those with a proximal JLO post‐TKA, although statistical significance was not observed. This is likely due to the relatively small sample size of our cohort, with a larger number of patients needed to analyze this outcome. An increased JLO would predispose to an increased medial bony resection and minimal lateral bony resection at the time of TKA, which in turn could affect the medial ligament tension leading to an increased chance of varus valgus laxity. This was evident by the fact that instability was the most common indication for revision surgery in patients with a proximal JLO post‐TKA. In such cases, technology‐assisted TKA could help to adjust component positioning to achieve more appropriate bony cuts and adequate ligament balancing. Surgeons should also be prepared to use an increased level of constraint to control the instability in such a situation. Similar to our results, Bae et al. [3] found that the lateral joint‐line inclination phenotype had an inferior long‐term survival rate after varus‐aligned TKA. They concluded that alignment and JLO both affected the long‐term survival rate of patients who underwent primary TKA.

In this cohort, patients who had a neutral JLO pre‐TKA (after HTO) and neutral JLO post‐TKA had better total WOMAC as well as stiffness and function sub‐scores as compared to those with a proximal JLO pre‐TKA, irrespective of a proximal or neutral JLO post‐TKA. Despite small numbers and wide CIs, these differences are clinically relevant as they considerably exceed the MCID [7]. In primary TKA, Konishi et al. [12] have shown that a post‐operative proximal JLO is a significant negative predictive factor for the Knee Society Score 2011 Score and Knee Injury and Osteoarthritis Outcome Score‐12 Score. This may be an important consideration while performing HTO to improve outcomes of consequent conversion TKA.

This study had several limitations. The retrospective nature of this study is associated with its inherent limitations. The cohort was small for formal survivorship analysis, especially in the subgroup analysis with wide CIs. This is mainly because conversion TKA following HTO is a relatively rare procedure. Considering this, a cohort of 110 TKAs is still a considerable number for this procedure. Additionally, the differences in WOMAC were significantly higher than the MCID and thus, our results are clinically relevant and can direct future research. While there is no single preferred method to measure JLO [24], we used the CPAK to determine JLO as it is the only method that includes both tibia and femur. Although it has limitations [10, 14, 18], CPAK is one of the most commonly used classifications to assess JLO and outcomes after TKA [2, 4, 9, 12]. This study did not separately study the causes of an increased JLO post‐HTO, which may have been secondary to overcorrection, hinge‐opening asymmetry or intra‐articular adaptation. However, the results of this study do suggest that increased JLO should be avoided at the time of HTO, and surgeons should modify their technique accordingly. These results are from a single centre with high volumes for both HTO and TKA and thus may not be reflective of different geographical regions or centres. The findings of our study will help direct future larger studies in a different population to confirm the effect of increased JLO after HTO on outcomes of conversion TKA.

## CONCLUSION

A proximal JLO created at the time of valgus‐producing HTO had an increased risk (OR = 4) of having a proximal JLO post‐TKA. While a proximal JLO after conversion to TKA did not show a statistically higher revision rate, maintaining a neutral JLO during HTO and at the time of subsequent TKA was associated with higher post‐operative clinical scores. However, further research in different and larger populations is needed to confirm these findings.

## AUTHOR CONTRIBUTIONS

**Brent A. Lanting**: Conceptualization; methodology; writing—review and editing; supervision. **Alan M. J. Getgood**: Conceptualization; methodology; writing—review and editing; supervision. **Adit R. Maniar**: Data curation; methodology; writing—original draft. **Nicola D. Mackay**: Data curation; methodology; writing—review and editing. **Lyndsay Somerville**: Methodology; formal analysis; writing—review and editing. **Robert Litchfield**: Methodology; writing—review and editing; supervision.

## CONFLICT OF INTEREST STATEMENT

Adit R. Maniar: Editorial Board—Arthroscopy. Robert Litchfield: Smith Nephew: Educational grants, royalties, consultant and Conmed: Educational consultant, ARC Medical Devices: Consultant and shareholder, Integrated Endoscopy: Consultant, Sensor Therapeutics: Consultant. Brent A. Lanting: Principal investigator grant—Smith and Nephew, Zimmer, DePuy; Institutional support—Smith and Nephew, Zimmer, DePuy and Stryker; Consultant—DePuy. Alan M. J. Getgood: Aesculap/B.Braun: Research support. American Journal of Sports Medicine: Editorial or governing board. Graymont Inc: IP royalties. International Society of Arthroscopy, Knee Surgery, and Orthopaedic Sports Medicine: Board or committee member. Knee Surgery, Sports Traumatology, Arthroscopy: Editorial or governing board. LinkX Robotics: Stock or stock Options. Ossur: Research support. Ostesys Robotics: Stock or stock Options. Precision OS: Stock or stock Options. Smart HTO: Stock or stock Options. Smith & Nephew: Paid consultant; Paid presenter or speaker; Research support. Spring Loaded Technologies Inc: Stock or stock Options. Surgical Health Innovations Corp: Stock or stock Options. The remaining authors declare no conflicts of interest.

## ETHICS STATEMENT

Approval was obtained from the Institutional Research Ethics Board (REB 124314).

## Data Availability

Data not available publicly due to ethical considerations.

## References

[1] Ackerman IN , Kemp JL , Crossley KM , Culvenor AG , Hinman RS . Hip and knee osteoarthritis affects younger people, too. J Orthop Sports Phys Ther. 2017;47(2):67–79. 10.2519/jospt.2017.7286 28142365

[2] Agarwal S , Ayeni FE , Sorial R . Impact of change in coronal plane alignment of knee (CPAK) classification on outcomes of robotic‐assisted TKA. Arthroplasty. 2024;6(1):15. 10.1186/s42836-024-00239-1 PMC10993496 38570879

[3] Bae K , Lee BS , Kim JM , Bin SI , Lee J , Kim D , et al. Effect of joint‐line obliquity on long‐term survivorship of total knee arthroplasty: a postoperative phenotype analysis. Knee Surg Sports Traumatol Arthrosc. 2024;32:3230–3238. 10.1002/ksa.12311 38895851

[4] Bertugli E , Zambianchi F , Batailler C , Bazzan G , Lustig S , Catani F . Change of CPAK class does not affect functional outcomes in robotic arm‐assisted total knee arthroplasty performed with functional alignment. Knee Surg Sports Traumatol Arthrosc. 2025;33(5):1773–1783. 10.1002/ksa.12561 39666596

[5] Birmingham TB , Primeau CA , Moyer RF , Bryant DM , Ma J , Leitch KM , et al. High tibial osteotomy for medial compartment knee osteoarthritis: a randomized trial with parallel preference arm. Ann Intern Med. 2025;178:1238–1248. 10.7326/ANNALS-25-00920 40720836

[6] Clark GW , Steer RA , Khan RN , Collopy DM , Wood D . Maintaining joint line obliquity optimizes outcomes of functional alignment in total knee arthroplasty in patients with constitutionally varus knees. J Arthroplasty. 2023;38(7 Suppl 2):239–244. 10.1016/j.arth.2023.04.004 37061140

[7] Clement ND , Bardgett M , Weir D , Holland J , Gerrand C , Deehan DJ . What is the minimum clinically important difference for the WOMAC index after TKA? Clin Orthop Relat Res. 2018;476(10):2005–2014. 10.1097/CORR.0000000000000444 PMC6259858 30179956

[8] Elbardesy H , McLeod A , Ghaith HS , Hakeem S , Housden P . Outcomes of double‐level osteotomy for osteoarthritic knees with severe varus deformity: a systematic review. SICOT‐J. 2022;8:7. 10.1051/sicotj/2022009 PMC8973300 35363133

[9] Franceschetti E , Campi S , Giurazza G , Tanzilli A , Gregori P , Laudisio A , et al. Mechanically aligned total knee arthroplasty does not yield uniform outcomes across all coronal plane alignment of the knee (CPAK) phenotypes. Knee Surg Sports Traumatol Arthrosc. 2024;32(12):3261–3271. 10.1002/ksa.12349 38984905

[10] Jenny JY , Baldairon F . The coronal plane alignment of the knee classification does not correlate with the functional knee phenotype classification. Knee Surg Sports Traumatol Arthrosc. 2023;31(9):3906–3911. 10.1007/s00167-023-07394-z 36947230

[11] Hernigou P , Duffiet P , Julian D , Guissou I , Poignard A , Flouzat‐Lachaniette CH . Outcome of total knee arthroplasty after high tibial osteotomy: does malalignment jeopardize the results when using a posterior‐stabilized arthroplasty? HSS J. 2013;9(2):134–137. 10.1007/s11420-013-9344-x PMC3757480 24426858

[12] Konishi T , Hamai S , Tsushima H , Kawahara S , Akasaki Y , Yamate S , et al. Pre‐ and postoperative coronal plane alignment of the knee classification and its impact on clinical outcomes in total knee arthroplasty. Bone Joint J. 2024;106–B(10):1059–1066. 10.1302/0301-620X.106B10.BJJ-2023-1425.R1 39348894

[13] Lingard EA , Katz JN , Wright RJ , Wright EA , Sledge CB , Kinemax Outcomes G . Validity and responsiveness of the Knee Society Clinical Rating System in comparison with the SF‐36 and WOMAC. J Bone Joint Surg Am. 2001;83(12):1856–1864. 10.2106/00004623-200112000-00014 11741066

[14] Loddo G , An JS , Claes S , Jacquet C , Kley K , Argenson JN , et al. CPAK classification cannot be used to determine segmental coronal extra‐articular knee deformity. Knee Surg Sports Traumatol Arthrosc. 2024;32(6):1557–1570. 10.1002/ksa.12168 38643399

[15] MacDessi SJ , Griffiths‐Jones W , Harris IA , Bellemans J , Chen DB . Coronal Plane Alignment of the Knee (CPAK) classification. Bone Joint J. 2021;103–B(2):329–337. 10.1302/0301-620X.103B2.BJJ-2020-1050.R1 PMC7954147 33517740

[16] Nakayama H , Schröter S , Yamamoto C , Iseki T , Kanto R , Kurosaka K , et al. Large correction in opening wedge high tibial osteotomy with resultant joint‐line obliquity induces excessive shear stress on the articular cartilage. Knee Surg Sports Traumatol Arthrosc. 2018;26(6):1873–1878. 10.1007/s00167-017-4680-x 28831525

[17] Primeau CA , Birmingham TB , Leitch KM , Willits KR , Litchfield RB , Fowler PJ , et al. Total knee replacement after high tibial osteotomy: time‐to‐event analysis and predictors. Can Med Assoc J. 2021;193(5):E158–E166. 10.1503/cmaj.200934 PMC7954572 33526542

[18] Şahbat Y , Chou TA , An JS , Gülağacı F , Ollivier M . CPAK classification detect the real knee joint apex position in less than half of the knees. Knee Surg Sports Traumatol Arthrosc. 2024;32(6):1548–1556. 10.1002/ksa.12175 38613184

[19] Song J , Koh D , Liow L , Chia SL , Lo NN , Yeo SJ , et al. Alignment prior to total knee arthroplasty in high tibial osteotomy patients has no effect on subsequent functional outcomes. J Orthop Surg. 2022;30(3):10225536221132052. 10.1177/10225536221132052 36250492

[20] Song JH , Bin SI , Kim JM , Lee BS . What is an acceptable limit of joint‐line obliquity after medial open wedge high tibial osteotomy? Am J Sports Med. 2020;48(12):3028–3035. 10.1177/0363546520949552 32941061

[21] Stoddart JC , Dandridge O , Garner A , Cobb J , van Arkel RJ . The compartmental distribution of knee osteoarthritis: a systematic review and meta‐analysis. Osteoarthritis Cartilage. 2021;29(4):445–455. 10.1016/j.joca.2020.10.011 33253887

[22] Sun X , Wang J , Su Z . A meta‐analysis of total knee arthroplasty following high tibial osteotomy versus primary total knee arthroplasty. Arch Orthop Trauma Surg. 2020;140(4):527–535. 10.1007/s00402-020-03333-6 PMC7109181 32002662

[23] Vaishya R , Bijukchhe AR , Agarwal AK , Vijay V . A critical appraisal of medial open wedge high tibial osteotomy for knee osteoarthritis. J Clin Orthop Trauma. 2018;9(4):300–306. 10.1016/j.jcot.2018.02.004 PMC6224694 30449975

[24] Xie T , van der Veen HC , van den Akker‐Scheek I , Brouwer RW . Assessment of joint line obliquity and its related frontal deformity using long‐standing radiographs. J Orthop. 2023;40:57–64. 10.1016/j.jor.2023.04.014 PMC10172862 37188146

